# Framing sustainable food production technologies with videos – data from an experiment with German consumers

**DOI:** 10.1016/j.dib.2024.110695

**Published:** 2024-07-06

**Authors:** Ursula Ploll, Nina Weingarten, Monika Hartmann

**Affiliations:** University of Bonn, Chair of Agricultural and Food Market Research, Nussallee 21, 53115 Bonn, Germany

**Keywords:** Consumer acceptance, Videos, Implicit attitude, Information framing, Agricultural innovation

## Abstract

This pre-registered dataset was generated to investigate the effect of framing on consumer acceptance of beneficial soil-microbes in tomato production. For this purpose, an online experiment was conducted with 754 consumers in Germany in 2022. A market research agency recruited participants from their online panel, and quotas for a representative sample of the adult German consumer population were applied. Participants were randomly assigned to one of the following three experimental groups: gain-framing, loss-framing or a control group. Each group received a short video clip with information about beneficial soil-microbes, in which either gain-framing, loss-framing or no framing was applied. The following constructs were surveyed before video exposure: demographics, tomato consumption frequency, attitudes towards conventionally produced tomatoes and subjective knowledge of soil-microbes. The items and constructs collected after video exposure were: purchase intentions, explicit and implicit attitude towards tomatoes produced with beneficial soil-microbes, organic consumption behavior and an evaluation of different food production methods. A single-target category implicit-association test (ST-IAT) was used to measure the implicit attitude, and all other constructs were measured through self-reported survey items. The main concept of interest is the acceptance of beneficial soil-microbes, which can be inferred from consumers’ intentions and attitudes after video exposure. The dataset can be used to analyze the effect of video-based communication on consumer acceptance. Moreover, the effect of video-based communication on other outcome variables can be analyzed, such as the evaluation of different production methods and other exploratory purposes. Further, it can be explored whether organic consumption behavior or socio-demographic statistics play a mediating role on the effect of information framing. This might be of relevance for other researchers to determine the potential of video-based communication to influence consumers’ acceptance of future innovations in the food sector.

**Table:** Specifications Table

Subject	Social and Personality Psychology
Specific subject area	Effects of information framing in video clips about agricultural innovations on attitudes towards and intentions to purchase the innovation.
Data format	Raw, partially analyzed
Type of data	Table
Data collection	An online panel was used for data collection. Quotas on age, gender, education and occupation were applied. Participants were randomly assigned to one out of three treatment conditions. Exclusion mechanisms incorporated technical requirements, comprehension items and consistency checks. Participants who answered the IAT too slowly and too quickly (c.f. improved algorithm by [1]) were excluded.
Data source location	Institution: University of Bonn City/Town/Region: Bonn Country: Germany
Data accessibility	Repository name: Open Science Framework (OSF) Data identification number: 10.17605/OSF.IO/N9XJ5 Direct URL to data: https://osf.io/n9xj5/
Related research article	Ploll, U., Weingarten, N., & Hartmann, M. (2023). Frame by frame, attitude by attitude–The effect of information framing in videos on consumers’ acceptance of sustainable food production innovations. *Journal of Environmental Psychology*, 92, 102185.

## 1 Value of the data

• The dataset can be used to evaluate the effect of information framing in video clips on consumer acceptance towards an agricultural innovation. The effects of video clips with (a) loss-framing, (b) gain-framing and (c) a neutral control condition about beneficial soil-microbes in agriculture can be compared. The mediating role attitudes have regarding the effect of framing on purchase intentions can also be explored.
• Researchers or other actors in the food sector, who are engaging with innovation diffusion or transformation processes, can benefit from gaining insights into the role and potential of video-based information framing and its effect on consumer acceptance. Actors in the food sector can specifically benefit from further insights to create more effective communication efforts or future information campaigns which aim to promote acceptance towards sustainable agricultural innovations.
• Researchers may explore whether subjective knowledge, organic consumption behavior or socio-demographic characteristics impact the effect of information framing on consumer acceptance. Thereby, researchers can further explore what additional aspects underlie or mediate the influence of information framing on acceptance.
• The effect of information framing on the evaluation of conventional production methods, organic production methods and production using beneficial soil-microbes can also be explored. It stands to question how the information framing impacted the evaluation of production methods other than the agricultural innovation of interest.

## 2 Background

This dataset was generated to investigate the impact of framing of sustainable food production technologies in videos on consumer acceptance of beneficial soil-microbes in tomato production. Nowadays, consumers are exposed to many forms of video-based communications. However, research on the effect of videos as a communication medium remains limited [2]. This dataset contributes to understanding the potential impact of video-based communication strategies. Thus, the aim of this dataset is to enable analyses on the effect of information framing applied in videos on consumer acceptance. To evaluate acceptance, data on intentions and implicit and explicit attitudes was collected. It can be assumed that information provision changes both implicit and explicit attitudes, but Gawronski and Bodenhausen [3] illustrated that this is not necessarily always the case. Empirical research has also shown that the mediating role of implicit and explicit attitudes requires further exploration [4]. Therefore, this dataset enables further investigation on this relationship.

The associated research article focuses on a conducted mediation analysis. This data article provides in-depth information about the source of the data and the process of data generation, which could support additional exploratory analysis.

Future research can expand on the (limited) generalizability by implementing similar and/or comparable research with other consumer populations outside of Germany. Researchers may further explore the usage of visual means to enhance the effectiveness of information framing.

Moreover, the data set enables further analysis of German consumers’ food shopping intentions with respect to sustainable innovations, for example, through a consumer segmentation based on socio-demographic variables.

## 3 Data Description

The data file is based on an online experiment among German consumers, and consist of 745 complete responses. The following data cleaning was conducted and exclusion measures were implemented: (1) Participants with a low frequency of tomato consumption were excluded. (2) After exposure to the video, participants’ comprehension was checked with three questions about the content of the video. (3) An attention check item was included in the intention measure matrix. (4) Participants who indicated that they had never come across beneficial soil-microbes before, but had a mean score on the subjective knowledge scale (ranging from -3 to +3) higher than 1, were excluded. (5) Participants who answered the IAT too slowly or too quickly were excluded following the improved algorithm by Greenwald et al. [1]. Consequently, trials exceeding 10,000 milliseconds and participants who answered in under 300 milliseconds in more than 10% of the trials were excluded. If participants encountered any technical problems and their answers to the IAT could not be recorded, they were also excluded. Thus, the dataset consists of only complete cases with no missing values. A potential social desirability bias was approached by ensuring a self-administrated anonymous data collection, by reimbursement after participation irrespective of participants’ answers and by including an indirect measure [5].

Short descriptions and descriptive statistics of the main variables in the dataset can be found in Table 1. A detailed description of each variable, the corresponding survey text and coded answer options can be found in the codebook in the OSF folder.

**Table 1:** Descriptive summary of main variables.

Short description	Variable name	N	Mean	SD	Min	Max
Mean score: purchase intention	int	745	1.49	1.18	-3	3
Implicit attitudes (D-score)	atti_impl	745	0.25	0.33	-0.84	1.01
Mean score: explicit attitude towards tomatoes produced with soil-microbes	atti_expl	745	2.14	1.00	-3	3

*Note(s):* SD = standard deviation, Min = lowest possible value, Max = highest possible value.

## 4 Experimental Design, Materials and Methods

### 4.1 Experimental design

The pre-registered experiment followed a between-subjects design. Participants were assigned to one out of three treatment groups by exposure to a video: a loss-framing video (*n* = 200), gain-framing video (*n* = 322) or control video (*n* = 223). The difference in sample sizes emerged due to the comprehension check after video exposure. Participant who answered one out of three comprehension questions incorrectly were excluded. Pre- and post-treatment measures were identical for all participants, with only the video comprehension items differing between the groups. See Table 2 for an overview of all elements included in the experiment.

**Table 2:** Overview of materials and descriptions.

Materials	Description
Tomato consumption	Frequency of tomato consumption. Low frequency of tomato consumption led to exclusion.
Demographics	Age, gender, education, occupation. Quotas were applied to all demographic measures.
Attitudes towards conventional tomatoes	Bipolar attitude scale. Order of items was randomised.
Subjective knowledge	Subjective knowledge scale of soil-microbes. Order of items was randomised.
Information treatment (video)	Exposure to video clip (one out of three), followed by three questions of comprehension. Wrong answers for one or more of the comprehension questions led to exclusion.
Purchase intention	Hypothetical purchase intention scale. Order of items was randomised.
Explicit attitudes*	Bipolar attitude scale. Order of items was randomised.
Implicit attitudes*	Single-target IAT.
Organic consumption behaviour	Frequency of organic consumption and percentage of organic groceries.
Evaluation of production methods	Evaluation of conventional production, production with beneficial soil-microbes and organic production. Evaluation related to their perceived (a) naturalness, (b) healthiness and (c) environmental-friendliness. Matrix items were randomised.

*Note(s):* The order in the table conforms to the sequence of items in the questionnaire.

⁎ Implicit and explicit attitudes were counterbalanced.

### 4.2 Video materials

Three different videos with the corresponding framing were created lasting between 1:43 and 1:55 minutes. Generally, the three video clips were structured and arranged in the same manner. The videos explained what soil-microbes are and how they can interact with a plant. In the gain-framing video, advantages due to microbial application for the environment and for human health were portrayed, while in the loss-framing video, disadvantages due to the absence of microbial application were depicted. The design of the control group did not correspond to a true control group, as it is not possible to communicate the overall same content without any reference to benefits and harms. Hence, the control video instead explained in more detail how soil-microbes work.

The voice-over narration communicated the verbal framing stimuli. To increase the salience of the framing, visual cues were integrated in the videos. In the loss-framing video, wherever suitable, the color red was used, while in the gain-framing video, the color green was used, and in the control group grey color tones were integrated. Sample screenshots of the three different videos can be seen in Table 3; the videos can be found in the OSF folder.

**Table 3:** Exemplary frames of the video clips.

Gain-framing video	Loss-framing video	Control video
		

### 4.3 Measures

The measures “subjective knowledge”, “explicit attitudes” and “purchase intention” were all measured using a 7-point Likert scale. To ensure consistency and facilitate comparison with the D-score measure for implicit attitudes, the range of these measures was adjusted to span from -3 to +3. Thereby, for all measures, a score of “0” denotes a neutral evaluation, positive scores indicate favorable evaluations, and negative scores represent unfavorable evaluations.

### 4.4 Subjective knowledge

Subjective knowledge was measured with four items that were adapted from Flynn and Goldsmith [6]. Answers were coded on a 7-point Likert scale, ranging from “strongly disagree” (-3) to “strongly agree” (+3). The order of items was randomised. The four items were the following:

• “I know quite a lot about beneficial soil-microbes,”
• “I do not feel very well informed about beneficial soil-microbes,”
• “Compared to most other people, I know a lot about beneficial soil-microbes,”
• “When it comes to beneficial soil-microbes, I really don't know much.”

Items two and four of the subjective knowledge scale were reverse-coded. The recoded variables and the mean score variable are in the dataset.

### 4.5 Explicit attitudes towards conventionally produced tomatoes

The evaluation of conventional tomatoes was measured on a 7-point semantic bipolar scale. Six items measuring participants’ attitudes were implemented based on Richetin et al. [7], where the “bad–good” bipolar scale was added. Finally, the following bipolar scales were implemented: “unhealthy–healthy”, “negative–positive”, “bad–good”, “unattractive–attractive”, “unpleasant–pleasant”, “unenjoyable–enjoyable”. The order of each bipolar item was randomised. The bipolar scales were coded between -3 and +3. A mean score variable was created from these items, which is also included in the dataset.

### 4.6 Video comprehension

A binary variable indicates whether the video was re-watched by participants. After exposure to the video, participants’ comprehension was checked with three questions about the content of the video. If participants failed to answer at least one of these three comprehension items, they were excluded from the survey. The first question asked for a definition of soil-microbes; two answer possibilities were provided: “Small living organisms in the soil” or “A small rock in the soil.” The second question asked how soil-microbes affect the tomato plant. Two answer possibilities were provided: “The tomato plant becomes more resistant” and “The tomato plant reaches inflorescence earlier.” The third question asked at what level or how farmers need to adjust their production when using or not using beneficial soil-microbes. The first answer dealt with changes in crop protection measures and the second answer with changes in mechanical soil cultivation. The framing of the answers in the third question differed slightly according to the experimental groups. As it is written here, the first answer is always the correct answer; for participants the order of answers was randomised.

### 4.7 Purchase intention

Purchase intention was measured with three items based on the intention scale by Fishbein and Ajzen [8]. Answers were coded on a 7-point Likert scale, ranging from “strongly disagree” (-3) to “strongly agree” (+3). The order of items was randomised, and a mean score variable was created. The three items were the following:

• “I intend to purchase tomatoes produced using beneficial soil-microbes,”
• “I plan to purchase tomatoes produced using beneficial soil-microbes,” and
• “I will try to purchase tomatoes produced using beneficial soil-microbes.”

### 4.8 Explicit attitude towards tomatoes produced using beneficial soil-microbes

The explicit attitude towards “tomatoes produced using beneficial soil-microbes” was measured with the same scale and items as the explicit attitude towards “conventionally produced tomatoes.” A mean score variable was created from all six items.

### 4.9 Implicit attitude

The implicit attitude towards “tomatoes produced using beneficial soil-microbes” was measured by conducting a Single Target-Implicit Association Test (ST-IAT) following Bluemke and Friese [9]. The programming of the IAT was implemented using an R script by following Carpenter et al. [10]. In this way, the ST-IAT could be integrated in the online survey. Through the IAT plug-in, necessary records are stored in the variables Q1-Q16. These variables were needed for the creation of the D-score, which is already calculated and stored in the dataset. The D-score of differences was calculated by implementing the improved D-score algorithm of Greenwald et al. [1]. The code for the D-score can be found in the analytic script. Four different sets of the IAT were randomly displayed to the participants: the hypothesis-compatible pairing, where “positive” and “tomatoes produced with soil-microbes” were displayed together, and the hypothesis-incompatible pairing, where “negative” and “tomatoes produced with soil-microbes” were displayed together. 20 trials were conducted for the practice blocks and 74 for the test block. The images and categories used are displayed in Table 4.

**Table 4:** Images and words used in ST-IAT.

Images used in IAT	Categories used in IAT	
“Tomatoes produced with soil-microbes”	“Positive”	“Negative”
	Healthy Attractive Pleasant Enjoyable Good	Unhealthy Unattractive Unpleasant Unenjoyable Bad

### 4.10 Organic grocery shopping

Frequency of organic purchases was measured on a 6-point ordinal scale. Participants had to indicate how often they had bought organic food in the last 3 months: (1) “More than once a week”, (2) “Once a week”, (3) “2 to 3 times per month”, (4) “Once a month”, (5) “Less than once a month”, and (6) “Never”.

Additionally, the percentage of regular food expenditures on organic food was measured on a 9-point scale: (1) “Less than 10 percent”, (2) “About 11-20 percent”, (3) “About 21-30 percent”, (4) “About 31-40 percent”, (5) “About 41-50 percent”, (6) “About 51-60 percent”, (7) “About 61-70 percent”, (8) “About 71-80 percent” and (9) “More than 81 percent”.

### 4.11 Evaluation of production methods

The evaluation of different production methods was measured using three matrix items where participants had to evaluate (a) organic production methods, (b) production using beneficial soil-microbes, and (c) production following conventional production methods. Participants had to indicate how they perceived each of these three production methods with regard to (a) naturalness, (b) healthiness, and (c) environmental-friendliness on a five-point scale. The answer scales for naturalness ranged from (1) “not at all natural” to (5) “extremely natural”, the answer scale for healthiness from (1) “not at all healthy” to (5) “extremely healthy”, and the answers for environmental-friendliness from (1) “not at all eco-friendly” to (5) “extremely eco-friendly”.

## Limitations

The three experimental groups consist of different group sizes. This difference is an outcome from the comprehension check after video exposure; when answering at least one out of three questions wrongly, participants were excluded from further survey participation.

Furthermore, the sample is based on German consumers, generalizability is thus limited to the German consumer population.

## Ethics Statement

Informed consent was obtained from all subjects before participation. The consent form can be found in the survey file with the supplementary materials. The study was approved by the ZEF Research Ethics Board (certificate number 9_ILR_21).

## CRediT authorship contribution statement

**Ursula Ploll:** Conceptualization, Methodology, Software, Data curation, Formal analysis, Writing – original draft, Writing – review & editing, Funding acquisition. **Nina Weingarten:** Conceptualization, Methodology, Writing – review & editing. **Monika Hartmann:** Conceptualization, Methodology, Writing – review & editing, Funding acquisition.

## Data Availability

data_consumer (Original data) (Open Science Framework (OSF)).
